# Supplementary material for: Resolving the full spectrum of human genome variation using Linked-Reads
Source: Genome Res. 2019 Apr;29(4):635–45. doi: 10.1101/gr.234443.118 (PMC6442396; doi:10.1101/gr.234443.118)
Supplement: Supplemental Material [file supp_gr.234443.118_Supplemental_longranger-2.2.zip › longranger-2.2.tar.bz2!/lr2.2-public/lib/stan/cmdstan/stan/doc/api/html/functions_func_t.html]

Stan: Class Members - Functions


|  |  |
| --- | --- |
|  | Stan  2.5.0  probability, sampling & optimization |


- Main Page
- Related Pages
- Namespaces
- Classes
- Files

- Class List
- Class Index
- Class Hierarchy
- Class Members

- All
- Functions
- Variables
- Typedefs
- Enumerator
- Related Functions

- a
- b
- c
- d
- e
- f
- g
- h
- i
- j
- k
- l
- m
- n
- o
- p
- q
- r
- s
- t
- u
- v
- w
- x
- z
- ~


All Classes Namespaces Files Functions Variables Typedefs Enumerations Enumerator Friends Macros Pages

### - t -

- T()
  : stan::mcmc::base\_hamiltonian< M, P, BaseRNG >
  , stan::mcmc::dense\_e\_metric< M, BaseRNG >
  , stan::mcmc::diag\_e\_metric< M, BaseRNG >
  , stan::mcmc::unit\_e\_metric< M, BaseRNG >
- tangent()
  : stan::agrad::fvar< T >
- tau()
  : stan::mcmc::base\_hamiltonian< M, P, BaseRNG >
  , stan::mcmc::dense\_e\_metric< M, BaseRNG >
  , stan::mcmc::diag\_e\_metric< M, BaseRNG >
  , stan::mcmc::unit\_e\_metric< M, BaseRNG >
- term\_grammar()
  : stan::gm::term\_grammar< Iterator >
- to\_column\_major()
  : stan::json::json\_data\_handler
- to\_var()
  : stan::agrad::OperandsAndPartials< T1, T2, T3, T4, T5, T6, T\_return\_type >
- to\_vec()
  : stan::io::var\_context
- transition()
  : stan::mcmc::adapt\_dense\_e\_nuts< M, BaseRNG >
  , stan::mcmc::adapt\_dense\_e\_static\_hmc< M, BaseRNG >
  , stan::mcmc::adapt\_diag\_e\_nuts< M, BaseRNG >
  , stan::mcmc::adapt\_diag\_e\_static\_hmc< M, BaseRNG >
  , stan::mcmc::adapt\_unit\_e\_nuts< M, BaseRNG >
  , stan::mcmc::adapt\_unit\_e\_static\_hmc< M, BaseRNG >
  , stan::mcmc::base\_mcmc
  , stan::mcmc::base\_nuts< M, P, H, I, BaseRNG >
  , stan::mcmc::base\_static\_hmc< M, P, H, I, BaseRNG >
  , stan::mcmc::fixed\_param\_sampler
- type()
  : stan::gm::expr\_type


---

[ Stan Home Page ]

*© 2011–2014,
Stan Development Team.*
